# Valproate-induced hypothyroidism in schizoaffective disorder

**DOI:** 10.1192/j.eurpsy.2022.1862

**Published:** 2022-09-01

**Authors:** J. Facucho-Oliveira, P. Espada-Santos, A. Fraga, N. Moura, C. Laginhas

**Affiliations:** 1 Hospital de Cascais, Psychiatry, Alcabideche, Portugal; 2 Hospital Egas Moniz, Psychiatry, Lisbon, Portugal

**Keywords:** valproate, Hypothyroidism

## Abstract

**Introduction:**

Valproate is widely used in the treatment of maniac and mixed episodes and is well known to be safe with side effects being mostly related to hepatic disorders and psychomotor retardation.

**Objectives:**

Raising attention to valproate-induced hypothyroidism that despite the increasing evidence tends to be neglected.

**Methods:**

Here, we report a case of a 55-year-old woman, with a previous diagnosis of schizophrenia, treated for many years with 200mg of zuclopenthixol triweekly and 2mg of risperidone daily. Patient developed a maniac episode characterized by elevated mood, sense of grandiosity, increased energy and psychomotor activity, disinhibition and insomnia. No laboratory abnormalities were detected and inpatient treatment was initiated with paliperidone up to 12mg/day and valproate 1000mg/day.

**Results:**

Patient showed progressive clinical recovery attaining full remission within 2 weeks. Despite the absence of clinical side effects and the valproate serum levels of 74.9μg/mL (range 50–100μg/mL), laboratory testing found progressive reduction F-T4 down to 0.45ng/dL (range 0.8–1.5 ng/dL) and a concomitant upregulation of TSH to 73.99mUI/L (range 0.55–4.8mUI/L). Thyroid autoantibodies and thyroid echography were negative. Considering that patient was previously medicated with risperidone, it was suspected that her hypothyroidism was caused by valproate. Normalization of thyroid function was observed after 21 days valproate withdrawal. Patient is currently being treated with 150 mg paliperidone (monthly) with no recurrence of mood or psychotic episodes and maintain normal thyroid function.

**Conclusions:**

Our case emphasizes the need for extended laboratory testing upon prescription of new pharmacological medications as severe analytic alterations can take place in the absence of immediate clinical manifestation.

**Disclosure:**

No significant relationships.

